# Genome wide association study of uric acid in Indian population and interaction of identified variants with Type 2 diabetes

**DOI:** 10.1038/srep21440

**Published:** 2016-02-23

**Authors:** Anil K Giri, Priyanka Banerjee, Shraddha Chakraborty, Yasmeen Kauser, Aditya Undru, Suki Roy, Vaisak Parekatt, Saurabh Ghosh, Nikhil Tandon, Dwaipayan Bharadwaj

**Affiliations:** 1 Genomics and Molecular Medicine Unit, CSIR-Institute of Genomics and Integrative Biology, New Delhi 110020, India; 2 Academy of Scientific and Innovative Research, CSIR-Institute of Genomics and Integrative Biology Campus, New Delhi - 110020, India; 3 Human Genetics Unit, Indian Statistical Institute, Kolkata - 700108, India; 4 Department of Endocrinology and Metabolism, All India Institute of Medical Sciences, New Delhi 110029, India

## Abstract

Abnormal level of Serum Uric Acid (SUA) is an important marker and risk factor for complex diseases including Type 2 Diabetes. Since genetic determinant of uric acid in Indians is totally unexplored, we tried to identify common variants associated with SUA in Indians using Genome Wide Association Study (GWAS). Association of five known variants in *SLC2A9* and *SLC22A11* genes with SUA level in 4,834 normoglycemics (1,109 in discovery and 3,725 in validation phase) was revealed with different effect size in Indians compared to other major ethnic population of the world. Combined analysis of 1,077 T2DM subjects (772 in discovery and 305 in validation phase) and normoglycemics revealed additional GWAS signal in *ABCG2* gene. Differences in effect sizes of *ABCG2* and *SLC2A9* gene variants were observed between normoglycemics and T2DM patients. We identified two novel variants near long non-coding RNA genes *AL356739.1* and *AC064865.1* with nearly genome wide significance level. Meta-analysis and *in silico* replication in 11,745 individuals from AUSTWIN consortium improved association for rs12206002 in *AL356739.1* gene to sub-genome wide association level. Our results extends association of *SLC2A9, SLC22A11* and *ABCG2* genes with SUA level in Indians and enrich the assemblages of evidence for SUA level and T2DM interrelationship.

Uric acid is a by-product of oxidation of purine. SUA levels have been used as biological marker for many disorders like gout, arthritis, kidney functions^1^,^2^, hypertension, metabolic disorders and type 2 diabetes^3^,^4^. Studies have established SUA as an important stake holder regarding health issues of particular population. Hence it creates a necessity to study factors affecting SUA level of a population.

The levels of uric acid in an individual is a combined result of genetic factors and multitude of life style related factors like food habit, exercise, work type and means of transportation^5^,^6^,^7^,^8^. Indians differ in their food habit, living style and genetic constitutions from other ethnic populations in the world^9^,^10^,^11^.

Genetic studies have established a heritability of 40–70% for SUA level suggesting stronger role of genetic factors in determining SUA level^12^. Major part of genetic factors contributing to the SUA level has not been well understood as few number of genetic studies have been performed in limited populations and most of them being of European ethnicity. GWAS conducted on Japanese, Chinese, African American and Amish populations have established association of loci in urate transporter genes like *SLC2A9*, *ABCG2*, *SLC22A1* and *SLC22A12*^13^,^14^,^15^,^16^,^17^,^18^,^19^. Large scale meta-analysis conducted by Asian Genetic Epidemiology Network (AGEN) has included some samples from the Singapore Indian Study (SINDI) and identified loci in transcription factor *MAF* for SUA level^20^. Another large scale meta-analysis conducted by Global Urate Genetics Consortium^15^ identified 18 new loci associated with SUA level near *TRIM46*, *INHBB*, *SFMBT1*, *TMEM171*, *VEGFA*, *BAZ1B*, *PRKAG2*, *STC1*, *HNF4G*, *A1CF*, *ATXN2*, *UBE2Q2*, *IGF1R*, *NFAT5*, *MAF*, *HLF*, *ACVR1B*-*ACVRL1* and *B3GNT4* genes. Their analysis in 8380 samples of Indian ancestry showed association of variants in *SLC2A9, ABCG2, SLC22A11, GCKR, SLC17A1* gene at genome wide significance level. None of these study subjects were living in India. Both of the studies include Indian subjects from different ethnicities including Dravidian samples. This reflects a lack of independent studies conducted on Indian subjects for SUA level globally despite several waves of GWAS for different phenotype in different population. There is no separate genetic epidemiological study for uric acid levels in Indian population till date. Hence, genetic study in Indian population becomes necessary and provides a unique opportunity to explore the population specific genetic factors affecting uric acid level related to Indians.

Some of the identified genetic loci associated with SUA levels are found to show interactions with several phenotypes like sex, age and Body Mass Index (BMI)^21^,^22^. A recent epidemiological study showed a stronger association of SUA level with impaired fasting glucose suggesting a complex relationship between uric acid pathophysiology and glucose level^23^. T2DM is a condition where glucose metabolism of the individual gets impaired along with several other biochemical and signaling pathways. Another report showed association of uric acid transporter gene *SLC2A9* variant rs1014290 with T2DM status^24^. This suggests a plausible inter-relation between SUA related genetic factors and T2DM. However, there is no concrete study till date investigating the interaction between SUA genetics and T2DM. The present study aims at identification of common variants associated with SUA levels by two-staged Genome Wide Association Study (GWAS) in 4,834 healthy Indians of Indo-European ethnicity living in Northern part of India. Further, it extends its interest to explore the variability in effect of identified GWAS variants under altered condition (T2DM).

## Results

### Genome wide association analysis of SUA

**Figure 1:**
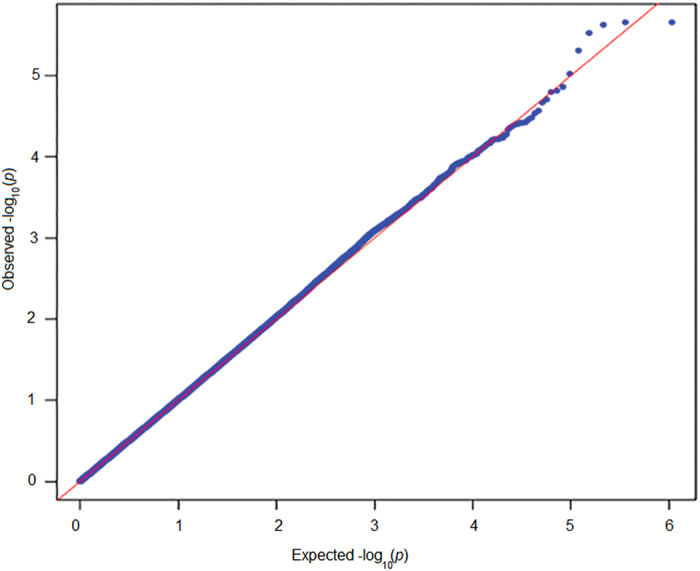
Quantile-quantile (QQ) plot for the calculated p-value in discovery phase. The -log10 of p-values observed for the association of SNPs in discovery phase genome wide association analysis under additive model adjusted for age, sex BMI, PC1 and PC2 (black symbols) are plotted against the theoretical -log10 p-values expected under the null hypothesis (red line). The genomic control inflation factor (λ) was estimated to be 1.06.

**Figure 2:**
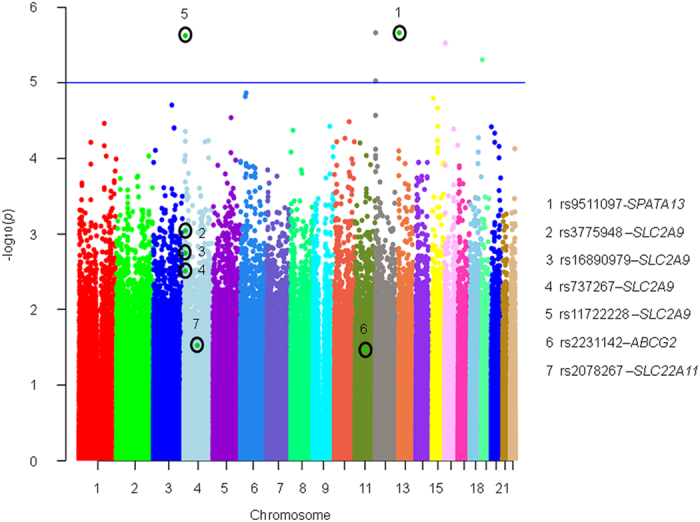
Manhattan plot for the SNPs associated with SUA levels in discovery phase. The -log10 p-values for association of genotyped SNPs are plotted as a function of genomic position (National Center for Biotechnology Information Build 37). The p-values were determined using logistic regression adjusted for age, sex, BMI, PC1 and PC2 in discovery phase analysis. Each chromosome (Chr) has been represented with a unique color.

After stringent quality control, we analyzed a total of 5,39,662 genetic markers in discovery phase for their association with SUA levels in 1,109 individuals using linear regression. A good agreement was observed between the theoretical p-value distribution and calculated p-values using QQ plot as shown in Fig. 1. The genomic inflation factor for the fitted model was calculated as 1.006 that indicates homogeneity of analyzed samples. *SPATA13* variants (rs9511097) were detected as most significant signal (Effect size = −16.35, p-value = 2.19 × 10^−6^) in discovery phase (Fig. 2). Along with the earlier known GWAS signals for SUA, SNPs with p-values<10^−4^ from discovery phase were genotyped in 3,725 Indian samples for replication phase. 4 SNPs associated with SUA level in 1^st^ phase had to be removed before final analysis in replication phase owing to their failure in quality control.

Meta-analysis was performed in 4,834 normoglycemic individuals and results yielded five SNPs in two different genes (*SLC2A9* and *SLC22A11*) associated with SUA levels at genome wide significance levels (Table 1, Fig. 2). Variant rs3775948 in *SLC2A9* gene showed most significant association with SUA levels in Indians (p-value = 1.7 × 10^−19^) that is in line with earlier findings in Japanese and African American population (Table 2)^13^,^14^. Another three detected variants in *SLC2A9* gene include rs16890979 (p-value = 2.62 × 10^−18^), rs11722228 (p-value = 7.34 × 10^−16^) and rs737267 (p-value = 2.71 × 10^−16^). The other genetic variant (rs2078267) attaining genome wide significant level was found to reside in *SLC22A11* gene (p-value = 3.26 × 10^−11^) (Table 1, Fig. 2). We also noticed almost genome wide association for missense variant rs2231142 (p-value = 7.82 × 10^−8^) in *ABCG2* gene (Table 1).

### Conditional analysis to examine independence of signals in *SLC2A9*

To examine independent association of signals in *SLC2A9*, conditional analysis of cumulative data (discovery and replication phase) was performed using additive model and result showed that rs3775948 (*SLC2A9*) as the lead SNP (Supplementary Table S1). Following conditional analysis for rs3775948, none of remaining three SNPs showed association at genome wide significance level but remained significant at sub-GWAS level (rs16890979, p-value = 1.078 × 10^−7^; rs737267, p-value = 1.86 × 10^−5^ and rs11722228, p-value = 1.64 × 10^−5^) (Supplementary Table S1). Results also indicated moderate linkage disequilibrium between rs3775948 and rs16890979 (R^2^ = 0.5).

### Status of earlier known GWAS signals

To examine the status of SUA associated variants across various populations, we compared effect size and association strength of known signals reported in GWAS catalogue among Indians and other major ethnic populations. Variants in *SLC22A12* gene (rs505802, P = 5.18 × 10^−6^) and *GCKR* gene (rs1260326, P=7.7 × 10^−4^ and rs780094, P=5.83 × 10^−3^) achieve sub-genome wide significance level in Indian population (Table 2). In addition, nominal association of variant rs1165205 in *SLC17A3* gene (P *= *0.03) with SUA level was detected in studied samples, whereas, markers in *PDZK1* (rs1967017), *ATXN2* (rs653178), *SLC17A1* (rs1165196), *BTF3P7-RREB* (rs675209) and *SLC16A9* (rs12356193) could not be replicated (Table 2).

Furthermore, difference in the effect size of genetic variants was also observed across population. Variants rs3775948 (*SLC2A9*, G allele) has been associated with decrease of uric acid level in Japanese population (Table 2) and African American population^14^, however it has higher effect size in Indians. Another non-synonymous coding SNP rs16890979 (A allele-Val253Ile) in exon 8 of *SLC2A9* gene has been associated with decrease in SUA levels in Chinese, European and Amish population^17^,^18^,^19^. The effect size of this variant in Indians is less than Caucasian and Amish population and higher than Chinese population (Table 2). The variant rs2078267 (A allele) in *SLC22A11* gene has higher effect size in Indians than in Caucasian population. Furthermore, *ABCG2* missense variant (rs2231142, Gln141Lys) has higher effect size in Indians than Caucasian, Chinese and Japanese population as shown in Table 2.

### Increase in number of GWAS signals with advent of type 2 diabetes

The effect of increasing sample size on the status of SUA associated genetic variants was examined. For this, meta-analysis of summary statistics obtained from association of T2DM subjects was conducted along with summary statistics from normoglycemic subjects. Analysis in a total sample of 5,911 individuals consisting of 4,834 normoglycemic subjects and 1,077 T2DM subjects improved the association of missense variant rs2231142 in *ABCG2* from 7.82 × 10^−8^ (Table 1) in normoglycemic subjects to 1.31 × 10^−10^ in combined sample (Table 3). Inclusion of T2DM samples in combined meta-analysis did not affect homogeneity of association (Table 3) and no difference in minor allele frequency (MAF) of rs2231142 was seen in normoglycemic and T2DM samples (Table 4).

### SUA variants have higher effect size in T2DM subjects

To unravel the possible inter-relation of SUA associated genetic variants with type 2 diabetes, we sought to examine whether these signals have different fate and effects in T2DM patients. We compared the effect sizes of signals that achieved GWAS level significance and were also significantly associated (p-value = 0.05) with SUA in T2DM subjects. Analysis revealed significant differences in the effect size of these variants between T2DM patients and normoglycemic subjects. Variants rs16890979 (A allele, d = −2.74) and rs737267 (A allele, d = −3.26) in *SLC2A9* genes possess higher effect size in presence of T2DM. Additionally, *ABCG2* gene variant rs2231142 (A allele, d = 1.71) was also reported to be associated with high SUA levels in T2DM patients (Table 4). Therefore, higher effect sizes of SUA genetic determinants in T2DM suggest possible interaction with T2DM pathophysiology. Joint analysis including T2DM patients and normoglycemic subjects adjusted for age, sex, BMI and diabetic status also confirmed the association of above six variants with SUA level at genome wide significance in Indian population. It also confirmed association of rs2231142 variant in *ABCG2* gene with SUA level in Indians as shown in Table 5.

Genotypes based on individual SNPs strongly correlate with average SUA level of subjects in Indians as shown in Supplementary Fig. S2. The cumulative effect analysis also revealed strong association of genetic score with SUA level (p-value = 2 × 10^−16^). Each additional allele causes a 6.45 unit increase in SUA level as shown in Supplementary Fig. S3.

### Enrichment analysis of the significant genes

Enrichment analysis was performed for the genes found significant in meta-analysis and result showed urate metabolic process as significantly enriched process in Indian population (Supplementary Table S2). Enrichment of six genes (*GCKR*, *SLC2A9*, *ABCG2*, *SLC22A11*, *SLC22A12* and *SLC17A3*) were observed against 11 genes (*PRPS1*, *SLC17A1*, *SLC22A11*, *SLC2A9*, *PNP*, *SLC22A12*, *SLC16A9*, *GCKR*, *SLC17A3*, *ABCG2* and *LRC16A)* present in human genome under urate metabolic process (accession number GO:0046415) with IMP (Inferred from mutant phenotype) category.

### Identification of novel signals in healthy subjects

Although our analysis in healthy subjects could not detect any novel genetic variants associated with SUA levels at genome wide significance levels, we have identified three novel loci (rs12206002, rs993701, rs1445305) in two different genes (*UTRN-AL356739.1, AC064865.1-RPL6P5*) at sub-genome wide significance levels (p-value<10^−5^, 10^−4^) (Supplementary Table S3). These loci could not be detected at genome wide levels even when our study was sufficiently powered (>98%) to detect these associations (Supplementary Fig. S1).

### Meta-analysis and *in-silico* replication

The meta-analysis of summary statistics for three novel loci (rs12206002, rs993701 and rs1445305) was performed with association data from AUSTWIN consortium. Meta-analysis improved the association status for variant rs12206002 from 8.83 × 10^−5^ to 7.23 × 10^−7^ (Supplementary Table S4). Heterogeneity in effect size of rs12206002 was observed across populations. We did not detect any improvement in the association of variant rs993701as it shifted to a meta-analysis p-value of 0.45 from 1.23 × 10^−4^ in Indian normoglycemic subjects. Association p-value for variant rs1445305 also increased from 9.85 × 10^−4^ to 0.21 after meta-analysis.

## Discussion

Present study is the first genome wide association study for SUA level in Indians. We found association of *SLC2A9*, *SLC22A11* and *ABCG2* gene variants at genome wide significance level (p-value < 10^−8^). This is the first study to report the differences in effect size of SUA associated genetic variants in *SLC2A9* and *ABCG2* in T2DM patients that suggests involvement of these gene variants in the alteration of uric acid levels in T2DM subjects. We also replicated variants in *GCKR* and *SLC17A1* gene at nominal significance levels. All these genes encode for transporter proteins. All significant genes explained nearly ~6% of the variance explained in uric acid level. This suggests contribution of other genetic, epigenetic and other factors in pathophysiology of uric acid levels in Indian population.

Studies have reported that *SLC2A9* is expressed in both kidney and liver of human and mice and is upregulated in diabetes mice^25^. The *SLC2A9* expression was found to be governed by p53 gene and is mediated by oxidative stress^26^. Oxidative stress play major and deterministic role in patho-physiology of T2DM and has been observed to be higher in T2DM patients than healthy controls^27^. The higher expression of *SLC2A9* in diabetic condition may be governed by higher oxidative stress in diabetics. In a recent study, Hurba *et al*. observed that there is no significant difference in transport activity of coding rs16890979 (Val253Ile) variant containing protein and wild type protein in *Xenopus* oocyte expression system^28^. The higher activity of *SLC2A9* in T2DM subjects compared to normoglycemics may be attributed to higher expression of total *SLC2A9* protein in T2DM condition.

**Figure 3:**
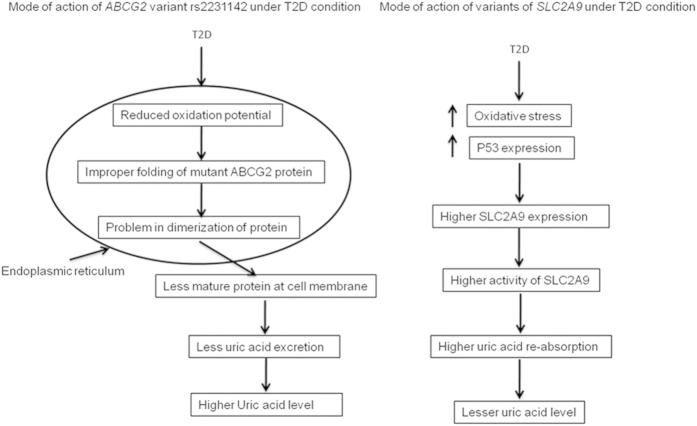
Mode of action of variants in *ABCG2* and *SLC2A9* genes under T2DM condition.

Similarly, another significant gene *ABCG2* is located at apical membrane of renal proximal tubule and is involved in excretion of uric acid in urine. The functional form of *ABCG2* protein present at cell membrane is homodimer glycosylated protein^29^,^30^. The homodimer formation requires proper folding and a disulphide bond formation between residues of two monomers in endoplasmic reticulum^29^. Reducing folding environment has found to induce improper folding in Q141K containing protein^31^. T2DM is a complex disorder where many biochemical processes, signaling cascades as well as hormone levels get altered. One of the major consequences of T2DM is alteration in redox potential in cellular compartments as well as extracellular spaces^32^. Study suggests that although there is an increase in oxidative environment of other compartments, the redox potential of microsomal vesicles from diabetic mice was found to shift towards more reducing end^32^. Because of these alterations of oxidative environment of microsomal vesicles towards comparatively more reducing environment in diabetic patients, more of the Q141K containing protein will be improperly folded and will be prone for proteosomal degradation. This will cause lesser protein trafficking, glycosylation and presence at the cell surface, finally lesser activity. This decrease in activity may increase the SUA level more in T2DM subjects than normoglycemics (Fig. 3). So far by our knowledge, there is no study to show evidence for T2DM interaction with *SLC2A9* and *ABCG2* genotypes. The observation regarding modulation in effect size of SUA associated variants in T2DM patients need to be confirmed in larger sample size and across different population. Possibility of any alternative mechanisms that modulates the effect of variants in *SLC2A9* and *ABCG2* genes under diabetic condition cannot be excluded.

Our study also showed the association of rs2078267 variant in *SLC22A11* gene at genome wide significance level in any population outside Caucasians for the first time. *SLC22A11* is a low affinity urate transporter and known to express in kidneys and placenta. *SLC22A11* has a role in the transport of multiple organic anions^33^.

Allele dosage analysis suggests that accumulation of studied variants in number may heavily contribute towards increasing SUA level in Indians.

Our study identified three novel variants near two different long non-coding RNA genes (*AL356739.1, AC064865.1*) at sub-genome wide levels (p-value < 10^−5^, 10^−4^) (Supplementary Table S3) in normoglycemics subjects. Stronger association of variant rs12206002 suggests its indispensable role to play in pathophysiology of SUA level in Indians and various other populations. Novel variants rs993701 and rs12206002 are 123kb and 130kb upstream of *AL356739.1* gene. Variant rs1445305 is 4.7 kb downstream of *AC064865.1* gene. Functions of these genes are only speculative and not confirmed. The variant rs1445305 is on an enhancer mark in K562 cell line. This suggests a possible functional role of these variants by altering the binding of transcription related protein.

In conclusion, our study extended the association of three uric acid transporter genes with SUA level to Indians at genome-wide significant level. It also indicated alteration in effect size of the genetic variants associated with SUA level with disease condition like T2DM. Our study further suggests involvement of both common as well as population specific genetic player to determine SUA levels. Enrichment of urate metabolic process in significant genes represents the contribution of uric acid transporter genes in determining SUA levels in Indians. These observations may have implications for further research into genetics of urate signaling in Indian population. These findings will also have possible impacts in pharmaceutical industry to understand the efficacy of urate lowering drug in altered condition like T2DM. The current information may also find relevance during treatment of patients with T2DM along with SUA related complications and may help in their better management.

## Methodology

### Study participants

All study participants in this study are the member of INdian DIabetes Consortium^34^. Samples are well characterized for anthropometric and biochemical parameters^34^ (Supplementary Table S5). These samples were enrolled in the study by conducting diabetes awareness camp organized in various parts of North India. Prior informed written consent was obtained from the study participants. The study was approved by the Human Ethics Committee of CSIR-Institute of Genomics and Integrative Biology and All India Institute of Medical Sciences research Ethics Committee. The study was conducted in accordance with the principles of Helsinki Declaration. Various anthropometric parameters like height, weight, waist circumference, hip circumference and biochemical parameters like total cholesterol, triglyceride, HDL and LDL were measured. The uric acid level was measured by enzymatic colorimetric method using COBAS Integra 400 plus (Roche Diagnostic, Mannheim, Germany).

### Discovery phase genotyping and quality control

Discovery phase samples were genotyped as a part of T2DM GWAS conducted in our laboratory using Illumina Human 610-quad bead chips^11^. Normoglycemic individuals used as control for T2DM GWAS were analyzed for current study. Genotype of these samples has been called by Gene call algorithm using Genome studio software. Strong quality control (both samples and SNPs) were performed on data. Samples were excluded based on call rate (<95%), heterozygosity (Samples with observed heterozygosity value 3 SD away from the mean heterozygosity were removed) and sex-discrepancy. Related samples were removed based on Identity-by-descent analysis (Pi-hat>0.1875). Cryptic relatedness for samples was calculated using 1,17,982 pruned SNPs. Pruning of the SNPs was done by applying –indep-pairwise command with a r^2^ 0.2 and window size of 50 using PLINK v1.07 (http://pngu.mgh.harvard.edu/~purcell/plink)^35^. Population outliers were detected using principal component analysis (http://www.complextraitgenomics.com/software/gcta/)^36^. First five principal components were used to detect population outlier samples. A total of 27 samples having eigenvectors 6 standard deviation away from the mean value were removed as outliers. SNPs with call rate (<97%), Hardy Weinberg equilibrium (p-value < 10^−5^) and MAF <0.01 were removed. We also removed SNPs in sex and mitochondrial chromosomes. Details for quality control steps have been given as Supplementary Fig. S4.

Samples with SUA level 3 standard deviations away of mean value were considered as outliers and were removed from the analysis. SUA levels were converted in to standard unit (μmol/L) and then inverse normalized using inbuilt command in R (http://www.r-project.org/). Association of SNPs with inverse normalized SUA levels was tested using linear regression model in PLINK. Sex, age, BMI and first three principal components were used as covariates in the model. To find the deviation of p-values obtained from an additive model a quantile-quantile (QQ) plot for –log (p-value) against theoretical p-value was plotted using qqman package in R.

### Validation phase genotyping and meta-analysis

Genetic markers from discovery phase having p-value < 10^−4^, in addition to known signals associated with SUA levels were taken for validation phase. The selected markers were genotyped along with other markers selected for ongoing GWAS for other quantitative traits in our laboratory using GoldenGate technology (Illumina San Diago, USA) and were analyzed in 3,725 Indians. A total of 204 samples (5.47%) were genotyped in duplicate, error rate of <0.01 was detected. Samples were removed having call rate <90%. SNPs with GenTran score <0.60, cluster separation score <0.4 and call rate <90% were excluded. We also excluded SNPs with minor allele frequency <0.01 and Hardy-Weinberg Equilibrium p-value <10^−5^. Samples with SUA level 3 standard deviations away from mean value were removed. SUA level was converted to standard unit and was inverse normalized before association. Association analysis was performed using linear regression model and model was adjusted for age, sex and BMI. Conditional analysis for loci in *SLC2A9* was done in the merged healthy samples using PLINK and model was adjusted for age, sex and BMI as covariates. Meta-analysis of the summary statistics of the stage 1 and stage 2 results was done by METAL (ttp:// www.sph.umich.edu/csg/abecasis/Metal/) using fixed effect inverse variance method^37^. All analyses have been done in healthy subjects if not stated otherwise.

We have also done joint analysis of both discovery phase and replication phase normoglycemic and T2DM subjects in seeking for association of some additional signals with SUA level. Joint analysis was done using linear regression and model was adjusted for age, sex, BMI and T2DM status.

### Enrichment analysis of the significant genes

To identify the functional contribution of the genes significantly associated with SUA levels, enrichment analysis was performed using GeneMANIA (http://www.genemania.org/)^38^. Enrichment for function of significant genes (p-value **<** 0.05) obtained during meta-analysis of normoglycemic subjects was done by searching for functions in GeneMANIA with pathway as network. Features with FDR corrected p-value < 0.05 were considered significant.

### *In-silico* Analysis of effect size of SUA associated variants in European ancestry subjects

The top three novel signals obtained from meta-analysis of stage 1 and stage 2 results in Indians were tested for association with SUA level using genome wide data from Australian Twin-Family Study (AUSTWIN) consortium in Australian samples of European origin^39^. AUSTWIN now includes 11741 adult participants (twins and their family members) with genotyping and uric acid results. Meta-analysis by combining the summary statistics for association in normolycemic subjects, T2DM subjects and AUSTWIN subjects was done by METAL. The effect sizes were converted to uniform unit by proper conversion factor before meta-analysis.

### Analysis of effect size of SUA associated variants in T2DM subjects

To determine possible difference in the impact of SUA associated genetic variants in diabetic conditions, we examined the effect size of markers associated with uric acid level at genome wide significance level in 1,077 T2DM subjects. We selected only those markers that were associated with SUA level (p-value<0.05) in both T2DM subjects and normoglycemic subjects. Data were analyzed separately for T2DM similar to normoglycemic subjects and effect size was estimated as mentioned above. Cohen’s d (d) value was calculated to compare the effect size. Cohen’s d was calculated as the ratio of difference of effect size between T2DM and normoglycemic divided by pooled variance of the two effect sizes.

### Combined risk score analysis

To find the cumulative affect of SUA increasing alleles for studied GWAS variant, effective unweighted genetic risk score was calculated. Genotypes were coded as 0, 1 and 2 based upon presence of zero, single or double SUA increasing allele in a subject. The effective genetic score was calculated as sum of scores for all six variants. To study the comparative enhancement in SUA level, subjects were divided into <2, 2–3, 4–5, 6–7, 8–9 and 10–11 genetic score containing groups. Mean value of SUA level was calculated and plotted against genetic score.

### Power calculation

Power of the study was calculated using Quanto software (http://hydra.usc.edu/gxe/) assuming additive genetic model for a range of allele frequencies from 0.001–0.5. Two tailed test at significance level of 0.05 with effect size ranging from 10.50–16.50 obtained from literature was used for power calculation and it was found that present study is sufficiently powered to detect association of genetic variants. A urate level of 308 μmol/L and standard deviation of 91 μmol/L was used. MAF for combined samples and calculated effect size from meta-analysis was used for final power analysis.

## Additional Information

**How to cite this article**: Giri, A. K. *et al*. Genome wide association study of uric acid in Indian population and interaction of identified variants with Type 2 diabetes. *Sci. Rep*. **6**, 21440; doi: 10.1038/srep21440 (2016).

## Supplementary Material

Supplementary Information

## Figures and Tables

**Table 1:** SNPs showing association with SUA at genome wide significance levels in meta-analysis.

Marker	CHR	BP	NearestGene	Al/A2	Discovery Phase		Validation Phase		Meta-analysis				
					Effect size (95% CI)	p-value	Effect size (95% CI)	p-value	Effect size (95% CI)	p-value	Dir	HetI Sq	Het-PVal
rs3775948*	4	9995182	*SLC2A9*	G/C	−11.96 (−18.95–−4.96)	8.41 × 10^−4^	−16.49 (−20.31–−12.67)	3.68 × 10^−17^	−15.45 (−18.80–−12.1)	1.70 × 10^−19^	−−	19.3	0.27
rs16890979*	4	9922167	*SLC2A9*	A/G	−12.53 (−20.2–−4.86)	1.41 × 10^−3^	−16.99 (−21.07–−12.92)	3.85 × 10^−16^	−16.01 (−19.61–−12.41)	2.62 × 10^−18^	−−	1.3	0.31
rs737267*	4	9934744	*SLC2A9*	A/C	−11.36 (−18.65–−4.08)	2.29 × 10^−3^	−15.35 (−19.28–−11.42)	2.51 × 10^−14^	−14.45 (−17.91–−10.99)	2.71 × 10^−16^	−−	0	0.35
rs11722228	4	9915741	*SLC2A9*	A/G	16.06 (9.365–22.76)	2.93 × 10^−6^	12.55 (8.831–16.27)	4.27 × 10^−11^	13.38 (10.13 −16.63)	7.34 × 10^−16^	++	0	0.37
rs2078267*	11	64334114	*SLC22A11*	A/G	−8.13 (−14.6–−1.66)	0.01	−11.27 (−14.83–−7.72)	5.65 × 10^−10^	−10.54 (−13.66–−7.43)	3.26 × 10^−11^	−−	0	0.41
rs2231142*	4	89052323	*ABCG2*	A/C	12.36 (0.43–24.3)	0.04	16.78 (10.22–23.35)	5.58 × 10^−7^	15.75 (10.01–21.50)	7.82 × 10^−8^	++	0	0.52

Chromosomal positions of SNPs are based on National Center for Biotechnology Information genome build 37. Alleles presented are indexed to the positive strand. Effect size was calculated with respect to the minor alleles. Direction was ++/−− if there was concordance between the discovery and validation phase and +−/−+ if there was discordance; Association results presented were obtained from genotyped data in 1,109 subjects from discovery phase and 3,725 subjects from validation phase.*Earlier reported variants taken in replication despite their higher p-value (>10^−4^) in discovery phase. Meta analysis has been done using METAL using fixed effect inverse variance method.

CHR: chromosome; BP: Base pair position; Dir.: direction; Het-P: p-value for heterogeneity in effect sizes in meta-analysis; Het-I Sq: Chi-square value for heterogeneity test.

**Table 2:** Comparison of effect size of known GWAS variants associated with SUA across different ethnic population.

NearestGene	SNP	Indians			Chinese				Japanese				Caucasians			
		MAF	Effect size	p-value	MAF	Effect size	p-value	Ref	MAF	Effect size	p-value	Ref	MAF	Effect size	p-value	Ref
SLC2A9	rs3775948	0.31 (G)	−15.45	1.70 × 10^−19^	NA	NA	NA	NA	0.59 (C)	0.18	2 × 10^−65^	13	NA	NA	NA	NA
SLC2A9	rs16890979	0.24 (A)	−16.01	2.62 × 10^−18^	0.02 (T)	−0.02	0.29	17	0.01 (T)	−0.18	3.07 × 10^−2^	13	0.29 (T)	−20.22	3.6 × 10^−189^	40
SLC2A9	rs737267	0.27 (A)	−14.45	2.71 × 10^−16^	NA	NA	NA	NA	NA	NA	NA	NA	0.31(T)	−0.88	3.0 × 10^−9^	41
SLC2A9	rs11722228	0.35 (A)	13.38	7.34 × 10^−16^	0.31 (T)	0.03	3.68 × 10^−6^	17	0.45 (T)	0.16	7.09 × 10^−24^	13	0.50 (T)	9.93	1.8 × 10^−75^	40
SLC22A11	rs2078267	0.41 (A)	−10.54	3.26 × 10^−11^	NA	NA	NA	NA	0.01	2.58	0.64	13	0.51 (T)	−4.34	9.0 × 10^−38^	15
ABCG2	rs2231142	0.08 (A)	15.75	7.82 × 10^−8^	0.29 (T)	0.05	1.2 × 10^−9^	17	0.31 (T)	0.12	1.62 × 10^−13^	13	0.11 (T)	10.29	3.1 × 10^−26^	40
SLC22A12	rs505802	0.41 (G)	7.36	5.18 × 10^−6^	0.23 (T)	−0.01	0.20	17	0.18 (T)	−0.23	1.00 × 10^−31^	13	0.30 (T)	−3.57	2.0 × 10^−9^	40
GCKR	rs1260326	0.23 (A)	6.24	7.70 × 10^−4^	NA	NA	NA	NA	NA	NA	NA	NA	0.41 (T)	4.40	7.0 × 10^−44^	15
GCKR	rs780094	0.24 (A)	5.10	5.83 × 10^−3^	0.44 (T)	0.01	0.01	17	0.43 (T)	0.04	5.12 × 10^−6^	13	0.42 (T)	2.97	1.4 × 10^−9^	40
SLC17A3	rs1165205	0.50 (A)	3.48	0.03	0.19 (T)	−0.01	0.14	17	0.16 (T)	−0.07	5.04 × 10^−4^	13	0.47 (T)	−5.35	4.0 × 10^−29^	18
SLC17A1	rs1165196	0.46(G)	−2.72	0.09	NA	NA	NA	NA	NA	NA	NA	NA	0.49 (G)	−6.25	5.0 × 10^−25^	16
RREB	rs675209	0.43 (G)	−2.55	0.12	NA	NA	NA	NA	NA	NA	NA	NA	0.27 (T)	3.64	7.0 × 10^−23^	15
ATXN2	rs653178	0.10 (G)	4.07	0.12	NA	NA	NA	NA	NA	NA	NA	NA	0.51 (T)	−2.08	7.0 × 10^−12^	15
SLC16A9	rs12356193	0.13 (G)	−1.60	0.50	NA	NA	NA	NA	NA	NA	NA	NA	0.83 (A)	4.76	1.0 × 10^−8^	40
PDZK1	rs1967017	0.33 (G)	−0.92	0.59	NA	NA	NA	NA	NA	NA	NA	NA	0.47 (T)	3.33	4.0 × 10^−8^	16

Effect sizes of current study (Indians) have been obtained from meta-analysis of normoglycemic subjects. Effect sizes of current study (Indians) and the source study are presented with respect to the minor allele as presented in MAF column of respective population. p-value indicates association with SUA levels. NA: not available; MAF: Minor allele frequency; Ref: Reference.

**Table 3:** Addition of T2DM subjects revealed addition SNPs associated with SUA at genome wide significance levels in meta-analysis.

SN	Marker	Gene	A1/A2	Discovery Phase		Validation Phase		Meta-analysis				
				Effect size (95% CI)	p-value	Effect size (95% CI)	p-value	Effect size (95% CI)	p-value	Dir	HetI Sq	Het-PVal
1	rs16890979	*SLC2A9*	A/G	−16.07 (−22.83–−9.316)	3.36 × 10^−6^	−17.15 (−21.18–−13.12)	1.02 × 10^−16^	−16.87 (−20.33–−13.40)	1.32 × 10^−21^	−−	0	0.79
2	rs737267	*SLC2A9*	A/C	−15.07 (−21.52–−8.614)	5.01 × 10^−6^	−15.67 (−19.55–−11.78)	3.61 × 10^−15^	−15.51 (−18.84–−12.18)	6.96 × 10^−20^	−−	0	0.88
3	rs3775948	*SLC2A9*	C/G	−10.34 (−16.45–−4.23)	9.27 × 10^−4^	−15.93 (−19.7–−12.15)	1.77 × 10^−16^	−14.39 (−17.60–−11.18)	1.58 × 10^−18^	−−	57	0.13
4	rs11722228	*SLC2A9*	A/G	12.66 (6.685–18.64)	3.44 × 10^−5^	12.68 (9.005–16.36)	1.60 × 10^−11^	12.67 (9.54–15.81)	2.21 × 10^−15^	++	0	1.00
5	rs2231142	*ABCG2*	A/C	21.00 (10.91–31.08)	4.67 × 10^−5^	16.75 (10.2–23.3)	5.60 × 10^−7^	18.01 (12.52–23.50)	1.31 × 10^−10^	++	0	0.49
6	rs2078267	*SLC22A11*	A/G	−6.61 (−12.37–−0.84)	0.02	7.72 (−11.25–−4.20)	1.78 × 10^−5^	−7.42 (−10.43–−4.41)	1.31 × 10^−6^	−−	0	0.75

Association results presented were obtained from genotyped data in 1,881 individuals including 1,109 healthy and 772 T2DM subjects from discovery phase and 4,030 subjects including 3,725 healthy and 305 T2DM subjects from validation phase. Effect size was calculated with respect to the minor alleles. Direction was ++/−− if there was concordance between the discovery and validation phase and +−/−+ if there was discordance. Meta analysis has been done using METAL using fixed effect inverse variance method.

Dir: direction; Het-P: p-value for heterogeneity in effect sizes in meta-analysis; Het-I Sq: Chi-square value for heterogeneity test.

**Table 4:** Comparison of association status of SUA associated variants in healthy subjects and T2DM subjects.

Marker	CHR	BP	NearestGene	Al/A2	Healthy Individuals			T2DM			
					MAF(A1)	Effect size (95% CI)	p-value	MAF(A1)	Effect size (95% CI)	p-value	Cohen’s d
rs16890979	4	9922167	*SLC2A9*	A/G	0.24	−16.01 (−19.61–−12.41)	2.62 × 10^−18^	0.23	−23.20 (−32.58–−13.81)	1.27 × 10^−6^	−2.74
rs737267	4	9934744	*SLC2A9*	A/C	0.27	−14.45 (−17.91–−10.99)	2.71 × 10^−16^	0.27	−22.62 (−31.56–−13.68)	7.07 × 10^−7^	−3.26
rs2231142	4	89052323	*ABCG2*	A/C	0.08	15.75 (10.01–21.50)	7.82 × 10^−8^	0.08	22.83 (8.19–37.47)	2.23 × 10^−3^	1.71

Association results presented in healthy individuals denote meta-analysis of 4,834 individuals from discovery and validation phase. Association results presented in T2DM subjects were presented for 1,077 diabetic individuals. Effect size was calculated with respect to the minor alleles. p-value shown here is obtained after meta-analysis of samples in discovery phase and replication phase. Cohen’s d was calculated as the ratio of mean difference of effect size between T2DM and healthy subject and pooled variance. Cohen’s d (d) value was calculated to compare the effect size. Cohen’s d was calculated as the ratio of difference of effect size between T2DM and normoglycemic divided by pooled variance of the two effect sizes. Chromosomal positions of SNPs are based on National Center for Biotechnology Information genome build 37.

CHR: chromosome; BP: Base pair position; MAF: Minor allele frequency.

**Table 5:** Association status of top signals achieving GWAS significance in joint analysis.

Marker	CHR	BP	NearestGene	A1/A2	Effect size (95% CI)	p-value
rs16890979	4	9922167	*SLC2A9*	A/G	−16.93 (−20.38–−13.49)	8.28 × 10^−22^
rs737267	4	9934744	*SLC2A9*	A/C	−15.59 (−18.90–−12.27)	3.82 × 10^−20^
rs3775948	4	9995182	*SLC2A9*	G/C	−14.06 (−17.25–−10.87)	7.68 × 10^−18^
rs11722228	4	9915741	*SLC2A9*	A/G	12.52 (9.41–15.63)	3.56 × 10^−15^
rs2231142	4	89052323	*ABCG2*	A/C	17.56 (12.12–23.01)	2.74 × 10^−10^
rs2078267	11	9934744	*SLC22A11*	A/G	−8.92 (−11.9–−5.95)	4.30 × 10^−9^

Association results for joint analysis performed in 5,911 (4,834 normoglycemic and 1,077 T2DM) individuals using genotype data for discovery and validation phase. Chromosomal positions of SNPs are based on National Center for Biotechnology Information genome build 37.

CHR: chromosome; BP: Base pair position MAF. CI: Confidence interval.
