# Supplementary material for: Genome wide association study of uric acid in Indian population and interaction of identified variants with Type 2 diabetes
Source: Sci Rep. 2016 Feb 23;6:21440. doi: 10.1038/srep21440 (PMC4763273; doi:10.1038/srep21440)
Supplement: Supplementary Information [file srep21440-s1.pdf]

Genome wide association study of uric acid in Indian population and interaction of identified variants with Type 2 diabetes

Anil K Giri[1,2], Priyanka Banerjee[1], Shraddha Chakraborty[1,2], Yasmeen Kauser[1,2], Aditya Undru[1,2], Suki Roy[1], Vaisak Parekatt[1], Saurabh Ghosh[3], *Nikhil Tandon[4], *Dwaipayan Bharadwaj[1,2,#5]

Supplementary Table S1: Conditional analysis of the SUA associated SNPs.

SNP	Meta-analysis			rs3775948			rs16890979			rs737267			rs11722228		
	**Gene**	**Effect size**	**p-value**	**Effect size**	**p-value**	**R^2**	**Effect size**	**p-value**	**R^2**	**Effect size**	**p-value**	**R^2**	**Effect size**	**p-value**	**R^2**
rs3775948	*SLC2A9*	-15.45	$1.699x10^{-19}$	NA	NA	1	-11.35	$2.19x10^{-9}$	0.5	-11.40	$6.042x10^{-9}$	0.22	-11.77	$1.6x10^{-9}$	0.21
rs16890979	*SLC2A9*	-16.01	$2.624x10^{-18}$	- 10.77	$1.078x10^{-7}$	0.50	NA	NA	1	-12.89	$3.56x10^{-3}$	0.45	-11.86	$7.1x10^{-9}$	0.17
rs737267	*SLC2A9*	-14.45	$2.712x10^{-16}$	-8.64	$1.86x10^{-5}$	0.22	-3.19	0.45	0.82	NA	NA	1	-10.15	$3.9x10^{-7}$	0.20
rs11722228	*SLC2A9*	13.38	$7.344x10^{-16}$	8.02	$1.64x10^{-5}$	0.21	8.76	$1.66x10^{-6}$	0.17	8.98	$1.28x10^{-6}$	0.2	NA	NA	1

Conditional analysis was performed in combined data from healthy subjects for significant SNPs in *SLC2A9* showing association with SUA levels during meta-analysis. Additive linear regression model adjusted for age, sex and BMI and SNPs genotypes was used for testing the associations.

Supplementary Table S2: Pathway analysis for the genes showing significant association with SUA levels in meta-analysis.

SN	**Feature**	**FDR corrected p-value**	**Genes in Networks**	**Gene in genomes**
1	Urate metabolic process	9.87×10^{-8}	6	11

Enrichment for function of significant genes (p-value < 0.05) in meta-analysis of control subjects was obtained by searching for functions in GENEMANIA with pathway as network. Features with FDR corrected p-value <0.05 were considered significant.

Supplementary Table S3: Novel signals associated with SUA level in Indian population.

					Discovery Phase		Validation Phase		Meta-analysis				
**Marker**	**CHR**	**BP**	**Nearest Gene**	**Al/A2**	**Effect size (95 % CI)**	**p-value**	**Effect size (95 % CI)**	**p-value**	**Effect size (95 % CI)**	**p-value**	**Dir**	**HetI Sq**	**Het-P Val**
rs12206002	6	145539467	*AL356739.1*	G/A	-11.04 (-18.32 - -3.75)	$3.06x10^{-3}$	-5.85 (-9.89 - -1.81)	$4.59x10^{-3}$	-7.07 (-10.60 - -3.53)	$8.83x10^{-5}$	--	32.9	0.22
rs993701	6	145546664	*AL356739.1*	A/G	-10.57 (-17.88 - -3.258)	$4.69x10^{-3}$	-5.82 (-9.86 - -1.78)	$4.80x10^{-3}$	-6.93 (-10.47 - -3.39)	$1.23x10^{-4}$	--	19.6	0.26
rs1445305	2	146047105	*AC064865.1*	A/G	-22.43 (-36.12 - -8.727)	$1.37x10^{-3}$	-7.83 (-15.64 - -0.01)	0.05	-11.41 (-18.20 - -4.62)	$9.85x10^{-4}$	--	69.6	0.07

Association results presented are obtained from genotyped data in 1,109 subjects from discovery phase and 3,725 subjects from validation phase. Chromosomal positions of SNPs are based on National Center for Biotechnology Information genome build 37. Alleles presented are indexed to the positive strand. Effect size was calculated with respect to the minor alleles; Direction was + if there was concordance between the discovery and validation phase and - if there was discordance; Earlier reported variants taken in replication despite their higher p-value $>10^{-4}$ in discovery phase.

CHR: chromosome; BP: Base pair position; Dir.: direction; Het-P: p-value for heterogeneity in effect sizes in meta-analysis; Het-I Sq: Chi-square value for heterogeneity test

Supplementary Table S4: *In-silico* replication and meta-analysis of novel SNPs rs12206002, rs993701 and rs1445305 in AUSTWIN cohort.

					Normoglycemic_meta-analysis		Normoglycemic and T2DM meta-analysis					Normoglycemic and T2DM and AUSTWIN meta-analysis				
**Marker**	**CHR**	**BP**	**Nearest Gene**	**Al/A2**	**Effect size (95 % CI)**	**P-value**	**Effect size (95 % CI)**	**P-value**	**Dir**	**HetI Sq**	**Het-P Val**	**Effect size (95 % CI)**	**P-value**	**Dir**	**HetI Sq**	**Het-P Val**
rs12206002	6	145539467	*AL356739.1*	G/A	-7.07 (-10.60 - -3.53)	$8.83x10^{-5}$	-5.57 (-2.27 - -8.86)	$9.16x10^{-4}$	-+	5.26	0.02	3.42 (2.07 - 4.77)	$7.23x10^{-7}$	-++	95	$2.44x10^{-9}$
rs993701	6	145546664	*AL356739.1*	A/G	-6.93 (-10.47 - -3.39)	$1.23x10^{-4}$	-5.35 (-8.64 - -2.05)	$1.48x10^{-3}$	-+	5.88	0.02	-0.52 (-1.87 - 0.83)	0.45	--+	87.3	$3.73x10^{-4}$
rs1445305	2	146047105	*AC064865.1*	A/G	-11.41 (-18.20 - -4.62)	$9.85x10^{-4}$	-10.7 (-16.95 - -4.43)	$8.91x10^{-4}$	--	0.29	0.59	-1.36 (-3.49 - 0.77)	0.21	---	79.9	$6.97x10^{-3}$

Effect size comparison and Meta-analysis of the 3 novel signals was done along with T2DM subjects and AUSTWIN data. Direction was ++/-- if there was concordance between the discovery and validation phase and +-/ -+ if there was discordance. Fixed effect inverse variance meta-analysis was done using METAL. For normoglycemic and T2DM meta-analysis result obtained from meta-analysis of discovery and validation phase normoglycemic subjects were analyzed with meta-analysis summary result T2DM subjects (772 discover phase samples and 305 validation phase samples).

Supplementary Table S5: Baseline characteristics of study participants in present study.

		Normoglycemic Subjects		T2DM Subjects	
**Feature**		**Discovery phase**	**Replication phase**	**Discovery phase**	**Replication phase**
Number(M/F)		1,109 (581/528)	3,725 (1982/1743)	772 (451/321)	305 (185/120)
Fasting Glucose (mg/dl) Median (IQR)	Male	87.3 (79.8 - 93.7)	90.6 (83.2 - 98.9)	305.6 (239.2 - 362.1)	331 (269.6 - 390.3)
	Female	87.0 (80.3 - 93.7)	89.1 (82.3 - 97.3)	268.6 (199.0-339.6)	293.2 (222.4-343.0)
BMI (kg/m^2) Median (IQR)	Male	23.4 (20.5 - 26.1)	25.3 (22.3 - 28.1)	23.6 (21.8 - 25.8)	26.8 (24.5 - 29.7)
	Female	25.4 (21.8 - 28.8)	27.3 (24.1 - 30.4)	26.7 (24.2 - 29.1)	29.2 (26.4 - 32.6)
Age (years) Median (IQR)	Male	50 (45 - 60)	51 (44 - 62)	54 (45 - 62)	56 (49 - 64)
	Female	50 (43 - 60)	49 (42 - 59)	52 (45 - 60)	55 (49 - 64)
Uric Acid (µmol/L) Median (IQR)	Male	331.9 (277.8 - 382.0)	352.0 (300.5 - 403.5)	136.0 (110.0 - 175.0)	143.8 (117.9 - 180.2)
	Female	253.5 (201.0 - 307.0)	272.3 (218.5 - 329.2)	143.0 (114.0 - 185.0)	153.9 (121.3 - 202.6)

N: Number of subjects; IQR: Interquartile range

Supplementary Figure S1: Statistical power of the study.

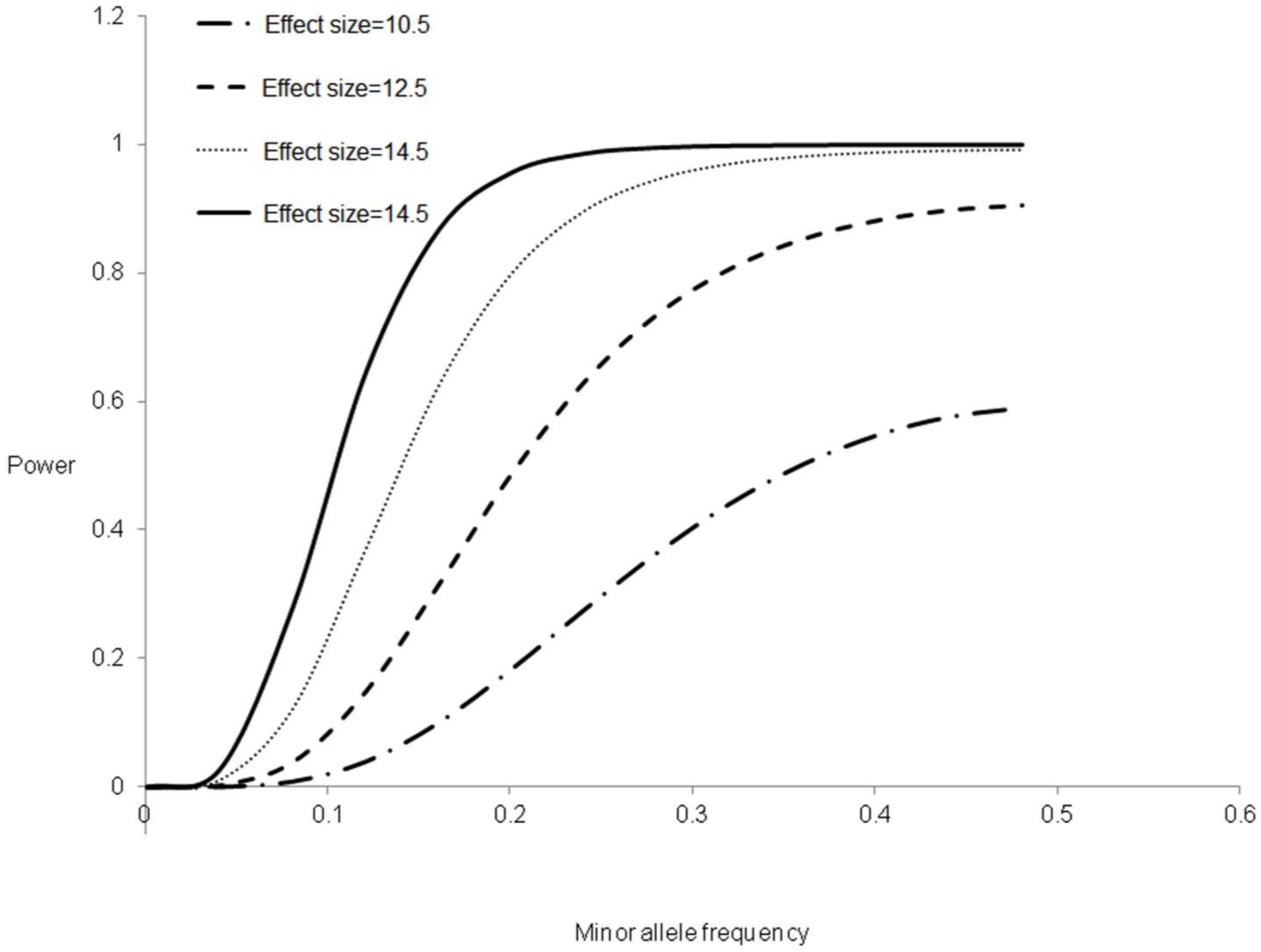

Statistical power of study for meta-analysis was calculated for allele frequencies ranging from 0.001 to 0.50 at different effect size from 10.50-16.50 assuming log additive model of inheritance at p-value=5×10^{-8}. Power has been plotted in y-axis for corresponding MAF in x-axis.

Supplementary Figure S2: Variation of SUA level according to different genotype for GWAS SNPs in normoglycemic subjects.

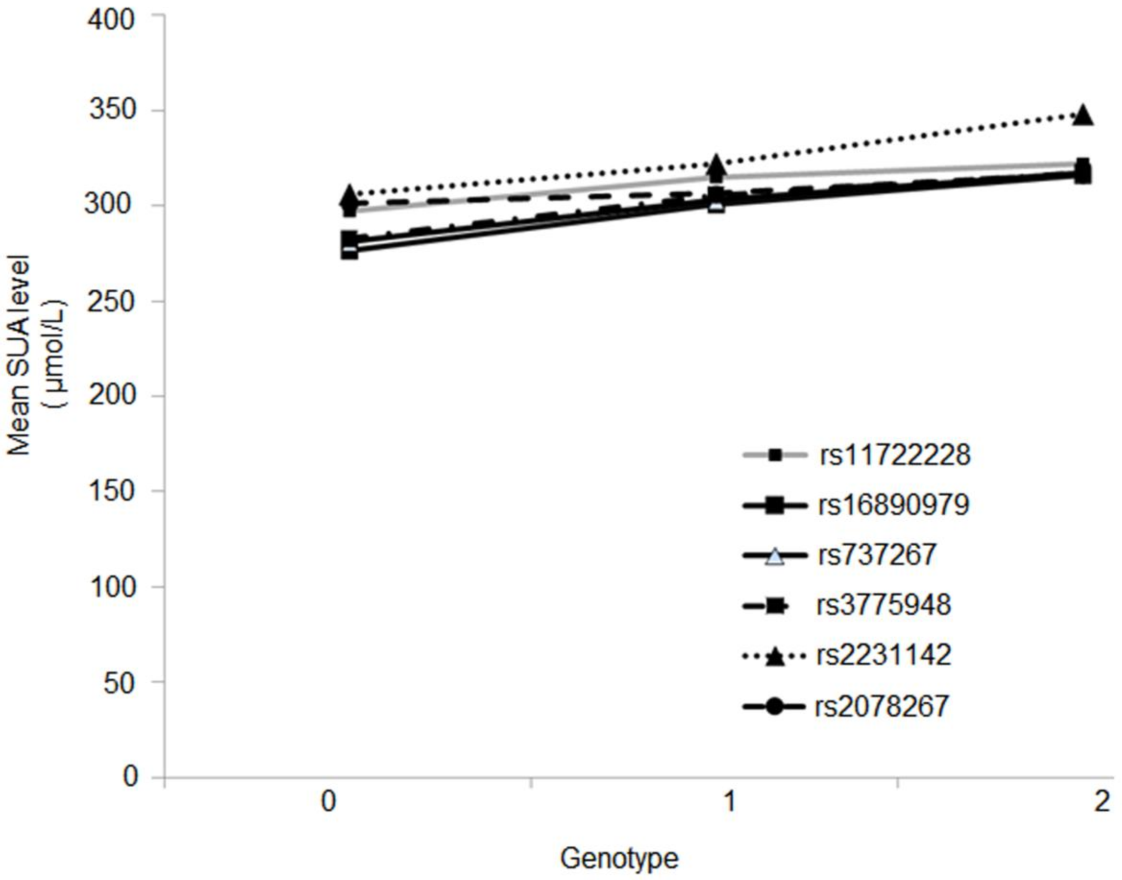

Mean of SUA level has been calculated and plotted for each genotype on y-axis for corresponding genotype on y-axis. Homozygote genotype with no SUA increasing allele, heterozygote genotype and homozygote genotype with two SUA increasing allele have been denoted by 0, 1 and 2 respectively.

Supplementary Figure S3: Bar plot showing increasing in mean SUA level with increase in unweighted risk score.

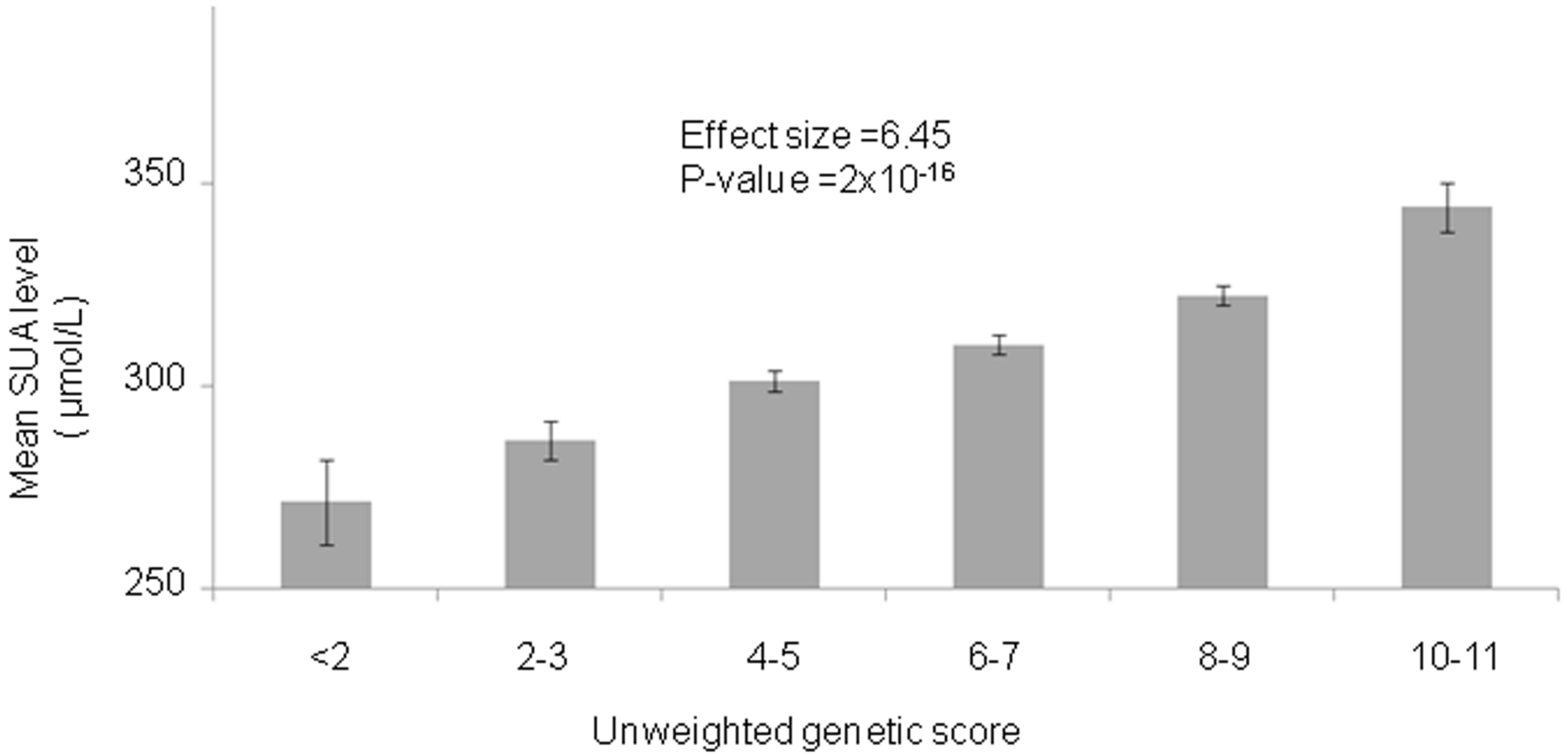

Combined risk score was calculated as sum of all SUA increasing allele in a sample. Samples were divided into different genetic score groups.

Mean of SUA level has been calculated and plotted for each group on y-axis for corresponding genetic score groups on x-axis.

Supplementary Figure S4: Details of quality control steps for samples and SNPs in discovery phase

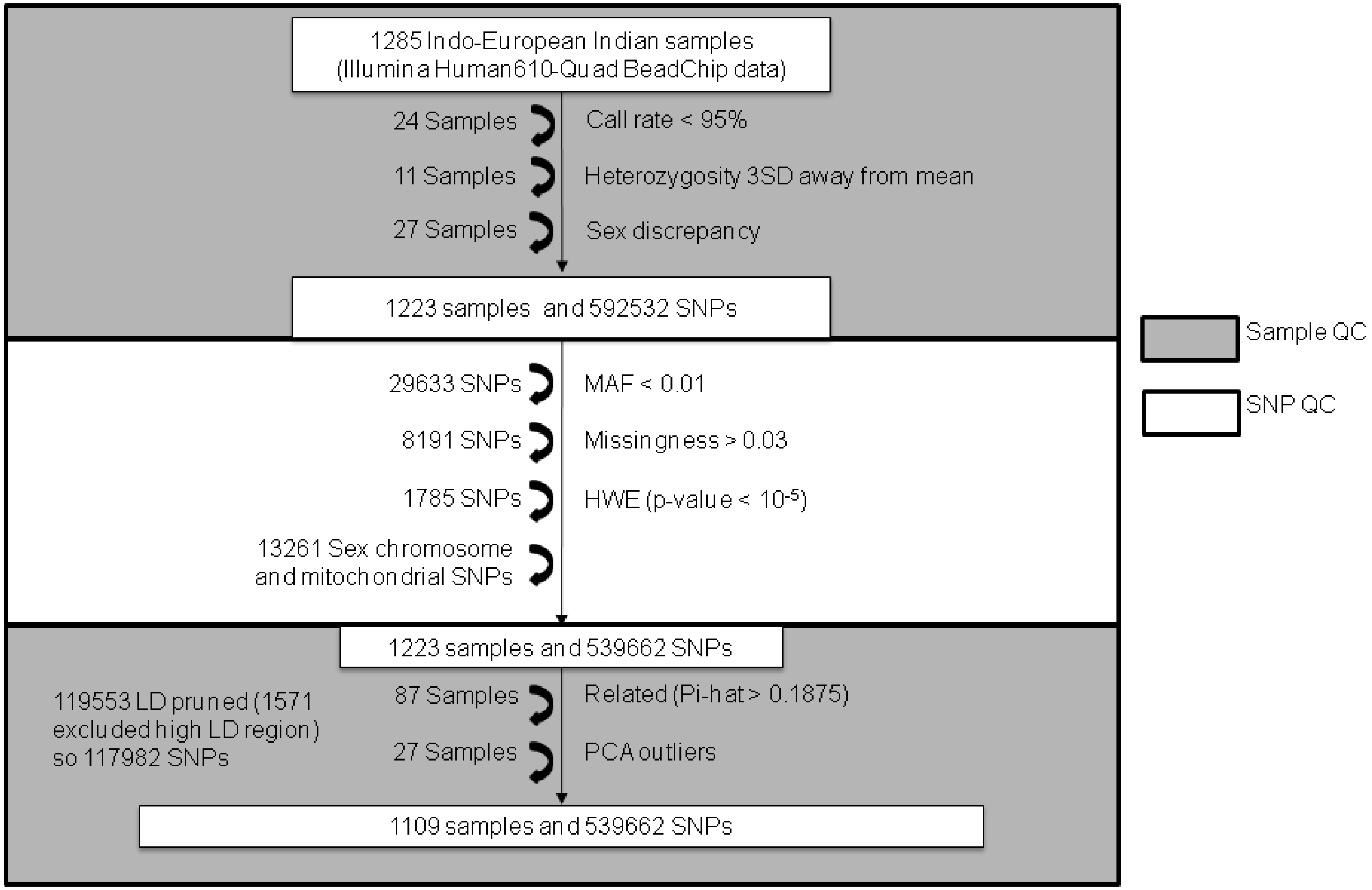

Samples with call rate <95%, heterozygosity, gender discrepancies and cryptic relatedness (pi_hat>0.1875) were excluded. SNPs with MAF <1%, call rate <97% for SNPs and deviating from Hardy-Weinberg equilibrium (p-value<1.0×10^{-5}) were excluded. Number of SNPs and samples excluded at each quality check step are presented.
